# Bowel Stimulation Before Loop Ileostomy Closure Using Probiotics: Study Protocol for a Randomized Controlled Trial at a Single Center

**DOI:** 10.2196/87422

**Published:** 2026-06-04

**Authors:** Kyeong Eui Kim, Sung Uk Bae, Seong Kyu Baek, Woon Kyung Jeong

**Affiliations:** 1 Department of Surgery, School of Medicine, Keimyung University Dongsan Medical Center, 1035 Dalgubeol-daero, Dalseo-gu, Daegu, 42601, Republic of Korea, +82-53-258-7879, +82-53-258-4170

**Keywords:** rectal neoplasms, rectal surgery, ileostomy, probiotics, low anterior resection syndrome

## Abstract

**Background:**

Low anterior resection syndrome (LARS) is a common functional problem after sphincter-preserving rectal cancer surgery and includes urgency, frequent bowel movements, clustering, and fecal incontinence. Diverting ileostomy may further disrupt the intestinal environment and alter the gut microbiota, potentially worsening bowel dysfunction after ileostomy closure. However, evidence remains limited on whether bowel stimulation with probiotics before ileostomy closure can improve postoperative bowel function and reduce LARS severity.

**Objective:**

This study aims to evaluate the safety, feasibility, and efficacy of probiotic bowel stimulation through the distal limb of a diverting ileostomy before ileostomy closure in patients with rectal cancer.

**Methods:**

This single-center randomized controlled trial will be conducted at Keimyung University Dongsan Medical Center, Republic of Korea. Eligible participants are adults aged 18‐80 years with clinical stage II or III rectal adenocarcinoma who completed neoadjuvant chemoradiotherapy and underwent laparoscopic or robotic low anterior resection with total mesorectal excision and diverting ileostomy, and who are scheduled for elective ileostomy closure. Participants will be randomly assigned in a 1:1 ratio to receive either 250 mL of normal saline with 4 g of Lacidofil or 250 mL of normal saline alone via the distal limb of the ileostomy once daily for 2 weeks before closure. The primary outcome is the LARS score 3 months after ileostomy closure. Secondary outcomes include postoperative complications, bowel recovery, stool habits, laboratory findings, and length of hospital stay. Analyses will primarily follow the intention-to-treat principle.

**Results:**

The study was approved by the Institutional Review Board of Keimyung University Dongsan Medical Center (DSMC-2024-03-016) and registered with the Clinical Research Information Service (KCT0011052). Recruitment is planned to begin in March 2026 and is expected to continue through March 2029. At the time of manuscript submission, the study is in the pre-enrollment stage, with no participants recruited and no data analysis performed. Results are expected to be published in 2029.

**Conclusions:**

This trial will provide prospective evidence on whether probiotic bowel stimulation before ileostomy closure is a safe and effective strategy for improving postoperative bowel function and alleviating LARS in patients undergoing rectal cancer surgery with diverting ileostomy.

## Introduction

Many patients with rectal cancer who underwent sphincter-preserving surgery experience symptoms such as increased stool frequency, fecal incontinence, irregular bowel movements, soiling, tenesmus, and other issues, collectively referred to as low anterior resection syndrome (LARS) [1,2]. LARS has a multifactorial etiology, including surgical injury to the anal sphincter, pelvic nerve damage, and anatomical changes within the pelvis. Risk factors for LARS include the resection level of the rectum, neoadjuvant chemoradiation, the method of anastomosis, use of a protective ileostomy, and the level of vessel ligation [1,3]. Several methods have been developed to alleviate the symptoms of LARS, including dietary modification, medication such as antispasmodics or constipating agents, pelvic floor rehabilitation, neuromodulation, and transanal irrigation. Despite these efforts, a definitive treatment for LARS remains elusive [2,4].

Diverting ileostomies are frequently performed in patients undergoing rectal cancer surgery, particularly in those at high risks of anastomotic leakage, such as those with a history of radiotherapy [5,6]. Diverting ileostomies provide the advantage of protecting the anastomosis and may reduce the severity of complications in the event of an anastomotic leak. However, the creation of a diverting ileostomy disrupts intestinal continuity, leads to mucosal hypotrophy, and alters the intestinal microenvironment, resulting in loss of intestinal peristalsis due to atrophy of intestinal smooth muscle [7]. Ileostomy closure is associated with postoperative morbidity, including ileus, bowel obstruction, wound complications, infectious complications, and anastomotic leakage, which may delay oral intake and prolong hospital stay [8].

Recent studies have demonstrated that the postoperative intestinal environment significantly impacts gut microbiota, leading to alterations in microbial community composition within the gastrointestinal tract [9,10]. Such changes may increase the proportion of harmful microbes, potentially contributing to severe complications [11,12]. Randomized controlled trials involving patients with colorectal cancer have shown that probiotics can adhere to the colon mucosa, thereby regulating gut microbiota and local immunity [13-15]. A recent study revealed that patients with severe LARS exhibited decreased microbial diversity, characterized by a higher Bacteroidaceae enterotype and reduced abundance of lactic acid-producing microorganisms, including *Bifidobacterium* and *Lactobacillus*, compared to those with mild LARS [15]. However, evidence regarding the role of probiotics in modulating gut microbiota after colorectal surgery and their effect on LARS remains limited [16,17]. Furthermore, the available evidence remains insufficient for routine clinical application in patients undergoing ileostomy closure after rectal cancer surgery [11,12,15,18]. Importantly, the current literature is limited by the small number of randomized or pilot trials, heterogeneity in patient populations, probiotic strains and formulations, timing and duration of administration, routes of delivery, and variation in primary endpoints

Therefore, this study aims to evaluate the safety, feasibility, and efficacy of administering probiotics via the distal limb of a diverting ileostomy before ileostomy closure in patients who underwent rectal cancer surgery. We hypothesize that probiotic bowel stimulation will improve the intestinal microenvironment, be well tolerated, and lead to improved postoperative bowel function, including a reduction in low anterior resection syndrome severity after ileostomy closure.

## Methods

### Study Design

The present study is designed as a single-center, randomized controlled clinical study conducted at Keimyung University Dongsan Medical Center, a tertiary referral hospital in Daegu, Republic of Korea. The study investigates the efficacy and safety of bowel stimulation with probiotics before ileostomy closure in patients with rectal cancer who received preoperative chemoradiation and underwent low anterior resection with ileostomy creation, compared to patients who did not receive probiotics. The protocol has been developed in accordance with the Standard Protocol Items: Recommendations for Interventional Trials (SPIRIT) checklist [19].

### Study Participants

Patients with rectal cancer who have undergone low anterior resection and a diverting ileostomy following chemoradiation and are scheduled for ileostomy closure will be enrolled. The investigator will provide detailed information about the study’s objectives, interventions, potential benefits, risks, and participant rights, allowing sufficient time for consideration. Patients will receive an informed consent form and may voluntarily sign it. Consent will also be obtained for reviewing medical records and collecting blood samples for recurrence assessment. Declining participation will not affect treatment, and patients may withdraw from the study at any time.

Inclusion criteria were as follows: (1) age 18‐80 years; (2) histologically confirmed rectal adenocarcinoma; (3) clinical stage II or III disease; (4) completion of neoadjuvant chemoradiotherapy; (5) prior laparoscopic or robotic low anterior resection with total mesorectal excision and creation of a diverting ileostomy; and (6) planned elective ileostomy closure after confirmation of anastomotic integrity.

Exclusion criteria were as follows: (1) suspected or confirmed inflammatory bowel disease; (2) evidence of anastomotic leakage, stricture, or distal obstruction precluding ileostomy closure; (3) medical contraindication to surgery; (4) severe immune-compromised status or uncontrolled systemic illness; and (5) inability or unwillingness to provide informed consent.

### Randomization and Allocation

Participants will be randomly assigned to either study group in a 1:1 ratio using a computer-generated allocation sequence. To ensure allocation concealment, the sequence will be secured in sequentially numbered, opaque envelopes, open only after participant enrollment. These envelopes will be prepared by a study staff member independent of patient enrollment and data collection. To reduce bias, surgeons performing the operations will remain blinded to group assignment until after anesthesia is initiated.

### Preoperative Workup

Approximately one month prior to surgery, patients will undergo preoperative evaluations, including an abdominopelvic computed tomography (CT) scan, a loopogram with rectal iodinated contrast, a digital rectal examination, and rectoscopy. During rectoscopy, any changes in the mucosa or anastomosis site will be meticulously documented. If such changes are observed, biopsies will be taken as part of standard care for patients with rectal cancer during oncological follow-up. A comprehensive preoperative clinical and anesthetic evaluation will also be conducted.

### Probiotics Administration

The patients will receive a 14-French Foley catheter, normal saline, and probiotics (Lacidofil®) containing *Lactobacillus rhamnosus* R0011 and *Lactobacillus acidophilus* R0052 bacterial culture (2×109 colony-forming units) in an outpatient setting. This formulation was considered appropriate for the present trial because Lactobacillus-based probiotics have been investigated in colorectal and other gastrointestinal settings as microbiota-modulating interventions, although evidence remains inconclusive regarding their effect on postoperative bowel function and LARS [20]. The probiotic dose was selected to ensure a practical and standardized daily regimen for outpatient administration while remaining aligned with the commercially available formulation and with doses that have been evaluated in prior studies using the same product or closely related probiotic preparations [21]. The intervention group will receive 250 mL of normal saline with 4 g of Lacidofil® administered via the distal limb of the ileostomy once daily for 2 weeks prior to ileostomy closure. The control group will receive 250 mL of normal saline without probiotics using the same method. The 250 mL of normal saline will be infused over 10‐15 minutes via a 14-French Foley catheter with the patient in a supine position. Adherence will be monitored through daily patient diaries and weekly outpatient visits. Patients will record abdominal symptoms, including pain, nausea, vomiting, diarrhea, and gas and stool passage during the 2-week period before ileostomy closure.

### Surgical Techniques and Surgeons

Three experienced surgeons, each having performed over 50 ileostomy closure procedures, will conduct the surgeries. All patients will consume two 200 mL cans of a complex carbohydrate drink 2 hours before surgery and fast thereafter, receiving a second-generation cephalosporin as prophylactic antibiotic therapy. Ileostomy closure will be performed as follows:

1. Position the patient in a supine position under general anesthesia.
2. Apply aseptic surgical drapes using alcoholic chlorhexidine and povidone-iodine solutions.
3. Make a circular incision around the ileostomy, dissecting in layers until the ileostomy is released from the aponeurosis.
4. Perform ileostomy closure with a side-to-side anastomosis using linear staplers.
5. Ensure hemostasis and close the wound layer by layer.

### Postoperative Management and Follow-Up

**Figure 1.**
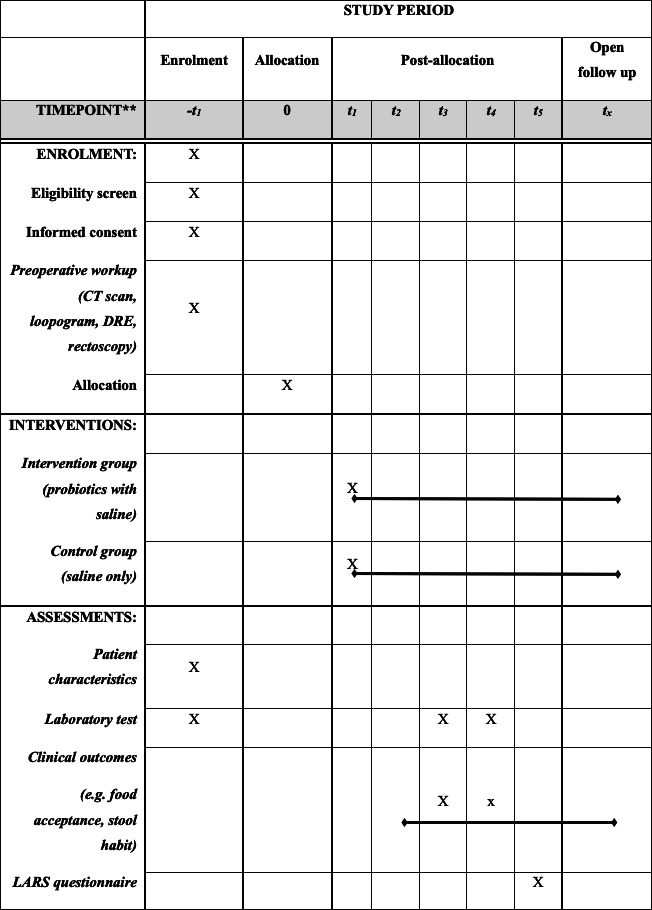
Standard Protocol Items: Recommendations for Interventional Trials (SPIRIT)-compliant schedule of enrolment, interventions, and assessments. CT: computed tomography; DRE: digital rectal exam; LARS: low anterior resection syndrome.

Oral intake will begin the day after ileostomy closure unless obstructive symptoms are reported. Hospital discharge will be determined by the surgeon based on adequate pain control without the need for intravenous or intramuscular analgesics, tolerance of a soft diet, independent ambulation, and absence of significant complications or other medical concerns. Patients will be followed for at least 3 months post-surgery, with visits at 1 week and 1 month. At each visit, patients will be assessed and questioned regarding food acceptance via patient-reported dietary intake logs, stool habits using the Bristol Stool Chart and daily frequency records, and laboratory tests. Three months post-surgery, patients will complete the LARS questionnaire in an outpatient setting. The detailed schedule of assessments is presented in Figure 1.

### Outcomes

The primary outcome is the LARS score at 3 months after ileostomy closure. The LARS score is a validated patient-reported outcome measure based on 5 items: incontinence for flatus, incontinence for liquid stools, bowel frequency, clustering of stools, and urgency. The total score ranges from 0 to 42, with higher scores indicating more severe bowel dysfunction. Scores are categorized as no LARS (0‐20), minor LARS (21-29), and major LARS (30-42). The questionnaire will be administered in the outpatient clinic at 3 months postoperatively.

The secondary outcomes include (1) postoperative complications within 30 days after ileostomy closure, including ileus, surgical site infection, anastomotic-related complications, readmission, and reoperation; (2) severity of postoperative complications, graded according to the Clavien-Dindo classification; (3) bowel recovery parameters, including time to first flatus, time to first stool passage, and tolerance of oral intake; (4) stool habits, including stool frequency and stool form assessed using patient records and the Bristol Stool Chart; (5) laboratory findings obtained during follow-up; and (6) length of postoperative hospital stay.

Patients will enter an open follow-up phase for up to 5 years for routine postoperative surveillance, monitoring oncologic outcomes and late complications through periodic clinical visits and imaging studies per institutional guidelines.

### Sample Size Calculation

The sample size calculation was based on the primary outcome, the LARS score at 3 months after ileostomy closure. Based on a previous study [21], we considered a 3-point between-group difference in LARS score to be clinically relevant. For the sample size estimation, the standard deviation was conservatively assumed to be 5 points. Using a 2-sided significance level of .05 and 80% power, 45 participants were required per group. Allowing a 10% dropout rate, the final target sample size was set at 100 participants (50 per group).

### Statistical Analysis

Results will be presented as means with standard deviations for continuous variables and as frequencies and percentages for categorical variables. The *χ*^2^ test or Fisher exact test will be used for categorical data, while the Student’s t-test or Mann-Whitney *U* test will be applied to continuous data. Logistic regression will be conducted for univariate and multivariate analyses. For adjusted analyses, prespecified covariates will include age, sex, body mass index, tumor location, clinical stage, neoadjuvant treatment history, operative approach, anastomotic level, and the interval between index rectal surgery and ileostomy closure. Exploratory subgroup analyses may be performed according to sex, age group, operative approach, and anastomotic level. Because the study is not powered for subgroup comparisons, these analyses will be interpreted cautiously. A *P* value <.05 will be considered statistically significant.

Analyses will be conducted on an intention-to-treat basis, including all randomized participants in their assigned groups, regardless of protocol adherence. A per-protocol analysis will be performed as a sensitivity analysis to assess result robustness. For missing data, multiple imputation methods will be employed, assuming data are missing at random. Sensitivity analyses will evaluate the impact of missing data on primary and secondary outcomes. Statistical analyses will be conducted using SPSS software (version 25; IBM Corp., Armonk, NY, USA).

### Data Collection and Monitoring

Data will be collected using a standardized case report form and entered into a Microsoft Access database. Collected data will include demographic characteristics, baseline clinical variables, perioperative findings, intervention adherence, patient-reported bowel symptoms, dietary intake logs, Bristol Stool Chart records, laboratory results, postoperative complications, and follow-up outcomes including the LARS questionnaire. Data accuracy will be monitored through double data entry and weekly audits.

Participant retention will be promoted through reminder calls and flexible outpatient visit scheduling. Regardless of findings, results will be submitted for publication in a peer-reviewed journal. A data monitoring committee is not required, as probiotics are classified as low-risk interventions per International Council for Harmonisation (ICH) Good Clinical Practice (GCP) guidelines and prior studies [12,15]. No interim analyses are planned, and the study will conclude upon data collection completion unless unexpected adverse events prompt a halt and review.

### Ethical Considerations

This study was approved by the Institutional Review Board of Keimyung University Dongsan Medical Center (IRB No. DSMC-2024-03-016). The study will be conducted in accordance with the ethical principles of the Declaration of Helsinki and applicable institutional requirements. Written informed consent will be obtained from all participants before enrollment and before any study-related procedures are performed. The investigator or designated research staff will explain the study objectives, procedures, potential risks and benefits, and participant rights, and sufficient time will be provided for questions and consideration.

Participant data will be d-identified before analysis and stored securely in a restricted-access setting. Access to identifiable information will be limited to the principal investigator and authorized research staff. No personally identifiable information will be disclosed in any publication or report arising from this study. Participants will not receive financial compensation for participation. However, participants who experience study-related harm or adverse effects will be managed and compensated in accordance with applicable institutional policies and regulations.

### Protocol Amendments

The protocol may be modified through agreement between the principal investigator and trial participants, with any changes requiring Institutional Review Board approval. Participants will be informed of amendments via written notification, and re-consent will be obtained if changes affect their participation.

### Reporting of the Study Results

Study results will be shared with investigators and patients, published in a peer-reviewed journal regardless of findings, with authorship following International Committee of Medical Journal Editors (ICMJE) guidelines. The full protocol will be publicly available via the Clinical Research Information Service (CRIS) registry, with deidentified data available upon reasonable request.

## Results

### Study Status and Timeline

The study protocol received approval from the Institutional Review Board of Keimyung University Dongsan Medical Center (IRB No. DSMC-2024-03-016) and was registered with the Clinical Research Information Service, Republic of Korea (KCT0011052). Participant recruitment is scheduled to begin in March 2026 and is expected to continue through March 2029. The target sample size is 100 participants, with 50 participants allocated to each study group. Final data collection for the primary endpoint will be completed after the 3-month follow-up of the last enrolled participant, and the main study findings are expected to be published in 2029.

### Recruitment and Protocol Implementation

As for manuscript submission, participant enrollment had not yet started because the study was in the pre-enrollment phase following ethics approval and trial registration. Therefore, no participants had been randomized, no baseline data had been collected, and no outcome analyses had been performed. No protocol amendments, deviations, or interim analyses had occurred before manuscript submission.

## Discussion

Probiotics have emerged as a pivotal intervention for bowel stimulation in patients undergoing ileostomy closure, a procedure often associated with gut microbiota imbalances due to diverting ileostomies, which can lead to an increase in pathogenic microorganisms and a reduction in beneficial lactic acid-producing bacteria, contributing to complications such as severe LARS [12,17]. By administering probiotics, specifically Lacidofil (containing *Lactobacillus rhamnosus* R0011 and *Lactobacillus acidophilus* R0052), this study aims to enhance gut microbial diversity and promote the colonization of beneficial microorganisms, such as *Lactobacillus* and *Bifidobacterium*, which are known to support intestinal motility, improve mucosal health, and strengthen local immunity, thereby fostering a healthier postoperative intestinal environment and potentially improving long-term outcomes for patients with rectal cancer [17,18]. The direct delivery of probiotics to the distal ileostomy limb, bypassing other digestive organs, represents a novel approach that enhances the intervention’s effectiveness in modulating the colonic microbiota compared to traditional oral administration, which may be subject to degradation in the upper gastrointestinal tract. This targeted delivery method, combined with a robust randomized controlled trial (RCT) design, well-defined inclusion and exclusion criteria (eg, excluding patients with inflammatory bowel disease or contraindications to ileostomy closure), and comprehensive follow-up assessments using standardized LARS scoring at 3 months post-surgery, ensures the reliability, reproducibility, and clinical relevance of the findings. The focus on LARS, a debilitating post-surgical complication that significantly impacts quality of life, underscores the study’s importance in addressing an unmet need in colorectal surgery.

Despite these strengths, the study has several limitations that warrant consideration. First, as a single-center trial, the results may not be fully generalized to populations with differing demographic characteristics, clinical settings, or surgical practices. To address this limitation, the trial employs clearly defined eligibility criteria, standardized surgical techniques performed by experienced surgeons, and rigorous data collection protocols to enhance reproducibility. Future multi-center trials with larger and more diverse populations will be essential to confirm external validity. Second, the intervention uses a single probiotic formulation (Lacidofil), which may limit the applicability of findings to other probiotic strains or combinations, as their efficacy and microbial interactions may vary. While this choice ensures consistency and minimizes confounding from strain heterogeneity, subsequent research could explore alternative formulations, dosages, or delivery regimens to optimize outcomes. Third, although all participating surgeons have substantial experience with ileostomy closure (>50 cases each), subtle variations in operative technique and postoperative care, as well as variability in patient adherence to postoperative protocols (eg, dietary intake logs and follow-up visits), could introduce minor biases. To reduce these effects, the study employs standardized perioperative protocols, surgeon blinding until anesthesia induction, and adherence monitoring through patient diaries and scheduled follow-ups. Finally, the follow-up period specified for the primary endpoint (3 mo) may not capture long-term effects of the intervention on bowel function and oncologic outcomes. However, an open follow-up phase of up to 5 years is incorporated to collect extended outcome data, and future studies with even longer observation and mechanistic analyses, such as microbiome profiling and immune modulation assessments, will be important to fully elucidate the sustained impact of probiotic administration.

The potential impact of this study extends beyond its immediate findings, as it contributes to the growing body of evidence supporting probiotics as a safe and effective adjunct in colorectal surgery. By targeting the distal ileum directly, this approach may set a precedent for innovative delivery methods in microbiota-based therapies, potentially influencing future guidelines for postoperative care in patients with rectal cancer. Furthermore, the study’s emphasis on patient-centered outcomes, such as quality of life through LARS score improvement, aligns with the increasing focus on holistic recovery in surgical research.

## Supplementary material

10.2196/87422 Checklist 1 SPIRIT 2013 checklist

## Abbreviations

ICMJE: International Committee of Medical Journal Editors
LARS: low anterior resection syndrome
SPIRIT: Standard Protocol Items: Recommendations for Interventional Trials

## References

[1] Rosen H, Sebesta CG, Sebesta C (2023). Management of low anterior resection syndrome (lars) following resection for rectal cancer. Cancers (Basel).

[2] Ryoo SB (2023). Low anterior resection syndrome. Ann Gastroenterol Surg.

[3] Christensen P, Im Baeten C, Espín-Basany E (2021). Management guidelines for low anterior resection syndrome - the MANUEL project. Colorectal Dis.

[4] Zhang R, Luo W, Qiu Y (2023). Clinical management of low anterior resection syndrome: review of the current diagnosis and treatment. Cancers (Basel).

[5] Dehni N, Schlegel RD, Cunningham C, Guiguet M, Tiret E, Parc R (1998). Influence of a defunctioning stoma on leakage rates after low colorectal anastomosis and colonic J pouch-anal anastomosis. Br J Surg.

[6] Marusch F, Koch A, Schmidt U (2002). Value of a protective stoma in low anterior resections for rectal cancer. Dis Colon Rectum.

[7] Zheng Z, Tang J, Hu Y, Zhang W (2022). Role of gut microbiota-derived signals in the regulation of gastrointestinal motility. Front Med (Lausanne).

[8] Luglio G, Pendlimari R, Holubar SD, Cima RR, Nelson H (2011). Loop ileostomy reversal after colon and rectal surgery: a single institutional 5-year experience in 944 patients. Arch Surg.

[9] Beamish EL, Johnson J, Shaw EJ, Scott NA, Bhowmick A, Rigby RJ (2017). Loop ileostomy-mediated fecal stream diversion is associated with microbial dysbiosis. Gut Microbes.

[10] Lin XH, Jiang JK, Luo JC (2019). The long term microbiota and metabolic status in patients with colorectal cancer after curative colon surgery. PLoS One.

[11] van Praagh JB, Luo JN, Zaborina O, Alverdy JC (2020). Involvement of the commensal organism *Bacillus subtilis* in the pathogenesis of anastomotic leak. Surg Infect (Larchmt).

[12] Shogan BD, Belogortseva N, Luong PM (2015). Collagen degradation and MMP9 activation by Enterococcus faecalis contribute to intestinal anastomotic leak. Sci Transl Med.

[13] Madrigal-Matute J, Bañón-Escandell S (2023). Colorectal cancer and microbiota modulation for clinical use. a systematic review. Nutr Cancer.

[14] An S, Kim K, Kim MH, Jung JH, Kim Y (2022). Perioperative probiotics application for preventing postoperative complications in patients with colorectal cancer: a systematic review and meta-analysis. Med Bogota Colomb.

[15] Kim MJ, Park S, Park JW (2023). Gut microbiome associated with low anterior resection syndrome after rectal cancer surgery. Sci Rep.

[16] Stephens JH, Hewett PJ (2012). Clinical trial assessing VSL#3 for the treatment of anterior resection syndrome. ANZ J Surg.

[17] Yoon BJ, Oh HK, Lee J (2021). Effects of probiotics on bowel function restoration following ileostomy closure in rectal cancer patients: a randomized controlled trial. Colorectal Dis.

[18] Emile SH, Garoufalia Z, Barsom S (2023). Systematic review and meta-analysis of randomized clinical trials on the treatment of low anterior resection syndrome. Surgery.

[19] Chan AW, Tetzlaff JM, Altman DG (2013). SPIRIT 2013 statement: defining standard protocol items for clinical trials. Ann Intern Med.

[20] Lee JY, Chu SH, Jeon JY (2014). Effects of 12 weeks of probiotic supplementation on quality of life in colorectal cancer survivors: a double-blind, randomized, placebo-controlled trial. Dig Liver Dis.

[21] Song HJ, Kim JY, Jung SA (2010). Effect of probiotic Lactobacillus (Lacidofil® cap) for the prevention of antibiotic-associated diarrhea: a prospective, randomized, double-blind, multicenter study. J Korean Med Sci.

